# Lack of Evidence for a Relationship Between the Hypothalamus-Pituitary-Adrenal and the Hypothalamus-Pituitary-Thyroid Axis in Adolescent Depression

**DOI:** 10.3389/fendo.2021.662243

**Published:** 2021-05-24

**Authors:** Raphael Hirtz, Lars Libuda, Anke Hinney, Manuel Föcker, Judith Bühlmeier, Jochen Antel, Paul-Martin Holterhus, Alexandra Kulle, Cordula Kiewert, Johannes Hebebrand, Corinna Grasemann

**Affiliations:** ^1^ Department of Pediatrics II, Division of Pediatric Endocrinology and Diabetology, University Hospital Essen, University of Duisburg-Essen, Essen, Germany; ^2^ Department of Child and Adolescent Psychiatry and Psychotherapy, University Hospital Essen, University of Duisburg-Essen, Essen, Germany; ^3^ Department of Exercise and Health, Institute of Nutrition, Consumption and Health Faculty of Natural Sciences, University Paderborn, Paderborn, Germany; ^4^ Department of Child and Adolescent Psychiatry, University Hospital Münster, Münster, Germany; ^5^ Department of Paediatrics I, Paediatric Endocrinology and Diabetes, University Hospital of Schleswig-Holstein, Christian-Albrechts-University, Kiel, Germany; ^6^ Department of Pediatrics, Division of Rare Diseases, St Josef-Hospital, and CeSER, Ruhr-University Bochum, Bochum, Germany

**Keywords:** mood disorder, depression, children and adolescents, hypothalamus-pituitary-adrenal axis, hypothalamus-pituitary-thyroid axis, cortisol, TSH (thyroid stimulating hormone)

## Abstract

In adults with major depressive disorder (MDD), a dysfunction between the hypothalamus-pituitary-adrenal (HPA) and the hypothalamus-pituitary-thyroid (HPT) axis has been shown, but the interaction of both axes has not yet been studied in adolescent major depressive disorder (MDD). Data from 273 adolescents diagnosed with MDD from two single center cross-sectional studies were used for analysis. Serum levels of thyrotropin (TSH), free levothyroxine (fT4), and cortisol were determined as indicators of basal HPT and HPA axis functioning and compared to that of adolescent controls by t-tests. Quantile regression was employed in the sample of adolescents with MDD to investigate the relationship between both axes in the normal as well as the pathological range of cortisol levels, considering confounders of both axes. In adolescent MDD, cortisol levels and TSH levels were significantly elevated in comparison to controls (*p* = <.001, *d* = 1.35, large effect size, and *p* = <.001, *d* = 0.79, moderate effect size, respectively). There was a positive linear relationship between TSH and cortisol (*p* = .003, *d* = 0.25, small effect size) at the median of cortisol levels (50^th^ percentile). However, no relationship between TSH and cortisol was found in hypercortisolemia (cortisol levels at the 97.5^th^ percentile). These findings imply that HPT and HPA axis dysfunction is common in adolescents with MDD and that function of both axes is only loosely related. Moreover, the regulation of the HPA and HPT axis are likely subjected to age-related maturational adjustments since findings of this study differ from those reported in adults.

## Introduction

The hypothalamus-pituitary-adrenal (HPA) and hypothalamus-pituitary-thyroid (HPT) axes are affected in a considerable proportion of patients with major depressive disorder (MDD) (e.g., 1, 2), and endocrine dysfunction of both axes is hypothesized to be closely related (e.g., 3, 4). While it is acknowledged that the HPA axis is a major endocrine cause of HPT axis dysfunction in adults, the functional relationship between both axes is not fully understood and has not been studied in MDD in children and adolescents so far.

In adults with MDD, a distinct pattern of thyroid hormone levels has been reported. While TSH and fT4 levels are mostly within the normal range of thyroid function parameters, TSH is decreased and ft4 increased when compared to controls (4). These observations have been related to two complementary mechanisms by which the HPA axis affects HPT axis functioning: a) impaired central regulation and b) altered peripheral thyroid hormone homeostasis. Regarding the central regulation of thyroid function (a), cortisol has been shown to stimulate thyrotropin-releasing hormone (TRH) secretion from hypothalamic cells *in vitro* (5), which is inhibited *in vivo* by feedback originating in the hippocampus (6). In MDD, however, elevated cortisol levels may result in hippocampal damage (7–9), thereby causing a loss of inhibitory feedback on TRH secreting neurons (6). The resulting increase in TRH secretion has been shown to prompt TRH receptor downregulation at the level of the pituitary gland due to chronic overstimulation (10), supported by the findings of elevated TRH levels in the cerebrospinal fluid (CSF) of depressed patients (11, 12) and a blunted thyrotropin (TSH) response to TRH (13). In addition to a potential modifying effect of cortisol on the central nervous system, elevated cortisol levels may also affect peripheral thyroid hormone homeostasis (b). Cortisol reduces the activity of the deiodinase type 2, responsible for the conversion of T4 to T3 (14). The resulting increase of T4 provides inhibitory feedback to the pituitary gland with a subsequent decrease in TSH secretion.

While the pathophysiological framework as outlined above is well-founded in theory, there are also conflicting findings from basic research, e.g., regarding TRH levels in the CSF of depressed patients (15–18) and from clinical studies. For example, no evidence of a substantial relationship between the HPT and HPA axis was detected in the so-far largest study to address their interplay (1). Thus, a dysfunctional relationship between the HPT and HPA axis in MDD may not constitute a homogenous phenomenon that generalizes to all patients. Instead, and considering the above outlined pathophysiological framework, the relationship between both axes may need to be examined in patients with a certain degree of endocrine dysfunction, that is, elevated cortisol levels (hypercortisolemia).

While all of the above-discussed findings pertain to studies conducted in adults, the relationship between the HPT and HPA axis in adolescent MDD has hardly been investigated but may differ from that in adults (19). The only study to address the relationship between the HPT and HPA axis in adolescents also included adults. Therefore, the effects of age and puberty on this relationship cannot be disentangled (20). However, and in contrast to studies relying exclusively on samples of adult patients, this study found a positive correlation between cortisol and TSH levels. Also, in adolescents with MDD, neither a blunted TSH response to TRH (13) nor an abolished TSH peak at midnight (21) - common findings in adults - have been replicated.

Considering that the relationship between the HPT and HPA axis in MDD is insufficiently understood, especially in children and adolescents, the present analysis was intended to provide insights into a potential relationship on the level of pituitary (HPT) and peripheral hormone functioning (HPT and HPA axis) in adolescent MDD based on data from two previously conducted studies.

Concerning the above-outlined pathophysiology regarding the relationship between the HPT and HPA axis, the following hypotheses were derived:

*H_1A_* ****: In MDD, mean cortisol levels are elevated and mean TSH levels are decreased.
*H_1B_* ****: In MDD, mean cortisol and mean fT4 levels are elevated.
*H_1C_* ****: In MDD, there is a negative correlation between cortisol and TSH levels.
*H_1D_* ****: In MDD, there is a positive correlation between cortisol and fT4 levels.
*H_2A_* ****: Only in adolescents with MDD and hypercortisolemia, there is negative correlation between cortisol and TSH levels.
*H_2B_* ****: Only in adolescents with MDD and hypercortisolemia, there is positive correlation between cortisol and fT4 levels.

## Methods

### Study Design and Participants

Data of the present study were derived from the baseline assessments of a two-armed parallel-group, double-blind RCT which investigated the effect of vitamin D deficiency (25(OH) vitamin D ≤ 12 ng/ml [equivalent to ≤ 30 nmol/l]; DRKS00009758) on depressive symptoms in psychiatric in- or daycare patients treated at the Department of Child and Adolescent Psychiatry, Psychosomatics and Psychotherapy Essen (LVR-Klinikum Essen), Germany (22). Additionally, we used data from a cross-sectional study focusing on the relationship between nutrition and psychiatric disorders. Since the setting as well as instruments of assessment of both studies were identical, data were pooled for analysis. Both studies were conducted in accordance with the Declaration of Helsinki and approved by the local Ethics Committee (No. 15-6363-BO).

Patients were eligible for inclusion if they were aged 11 to 18.9 years. Exclusion criteria were a concurrent diagnosis of severe somatic disease and/or intellectual disabilities (IQ < 70) (23). For the present analysis, all patients with a diagnosis of MDD, complete information on either variable of interest, with the exception of information on in- and outpatient status, and without levothyroxine treatment were considered (N = 273).

Primary data from the norming sample (N = 573) of the cortisol assay used in the present study served as reference for cortisol levels in MDD. In short, the norming sample was based on leftover blood draws before minor surgery or for the exclusion of endocrine and other diseases in pediatric subjects with no evidence of active disease upon comprehensive evaluation (24, 25).

Moreover, Siemens (Healthineers, Germany) provided aggregated data on age- and sex-specific reference ranges for TSH (N = 348) and fT4 (N = 319), established according to international standards (CLSI guideline EP28-A3c). Further details regarding the norming sample are provided by the package insert.

### Diagnostic Instruments and Questionnaires

The diagnosis of MDD was established either *via* the semi-structured interview ‘Schedule for Affective Disorders and Schizophrenia for School-Aged Children - Present and Lifetime Version’ (K-SADS-PL) according to DSM-IV ^(19)^ (93.8% of patients) or *via* clinical assessment according to ICD-10 (6.2%) when no K-SADS-PL was performed.

Depression severity was determined by the Beck Depression Inventory II (BDI-II), a self-reported questionnaire that records the severity of depressive symptoms according to DSM-IV diagnostic criteria for major depressive disorder (MDD) over the past two weeks by 21 items (26). Answers are scored on a 4-point Likert scale (0-3), with higher scores indicating a greater degree of depression (26). A total score is calculated by summing the score on each item, and the result is classified to indicate mild (BDI-II 14-19 points), moderate (20–28), or severe (29–63) depressive symptoms (26).

Additionally, and amongst others, covariates known to affect HPT and HPA axis functioning, including the intake of medication [e.g., psychotropic medication (27), levothyroxine, combined oral contraceptives (COC) (28)] and health-related behavior such as smoking (25) were recorded on admission.

### Anthropometric Measures

Patients were subjected to a physical examination upon admission, including an assessment of body height and body weight. Height was determined in an upright posture to the nearest 0.1 cm using a wall-mounted stadiometer. Body weight was measured in underwear by an electronic scale to the nearest 0.1 kg. BMI was determined by the ratio of weight in kg and the height in meters squared (kg/m^2^). To consider effects of age and sex, BMI was z-transformed according to percentile charts for German children and adolescents (29) [RefCurv 0.4.4, https://refcurv.com (30)].

### Laboratory Studies

Blood samples were obtained from an antecubital vein in the early morning (6:30 to 8:00 am) after an overnight fast and transferred within one hour after sampling to the laboratory of the University Hospital Essen for analyses. Serum and plasma aliquots were stored at -80°C until steroid metabolome analysis by liquid-chromatography tandem mass spectrometry (LC-MS/MS) (24, 25). In brief, for LC-MS/MS analysis, the stored sample aliquots the calibrator and controls aliquots were combined with the internal standard mixture to monitor recovery. All samples were extracted using Oasis MAX SPE system Plates (Waters, Milford, MA, USA). LC-MS/MS was performed using a Waters Quattro Premier/Xe triple-quadrupole mass spectrometer connected to a Waters Acquity (Waters, Milford, MA, USA; **Table 1** for details on assays).

**Table 1:** Assays and their performance characteristics.

Parameter	Assay system	Assay type	Intra-assay variation	Total assay variation	Detection range
TSH	Siemens^*1^ ADVIA Centaur	CLIA	<4.3%	<6.7%	0.008 to 150 mIU/ml
fT4	Siemens ADVIA Centaur	CLIA	<4.6%	<4.6%	0.1 to 12 ng/dl
TPO-AK	Siemens IMMULITE 2000	ECLIA	<6.3%	<7.2%	5 to 1000 IU/ml
25(OH)-vitamin D	Siemens ADVIA Centaur	CLIA	<5.3%	<11.9%	10.5 to 375 nmol/l
cortisol	Waters^*2^ Acquity UPLC System	LC-MS/MS	<9.7%	<12.9%	0.1 to 200 nmol/l

(E)CLIA, (electro-)chemiluminescent immunoassay; LC-MS/MS, liquid chromatography-tandem mass spectrometry. ^*1^ Siemens Healthineers, Erlangen, Germany. ^*2^ Waters, Milford, USA.

### z-Transformation

Based on age- and sex-specific reference ranges for TSH and fT4 provided by Siemens, TSH and fT4 levels were z-transformed by employing RefCurv 0.4.4 (30).

Relying on primary data of the norming sample of the LC-MS/MS cortisol assay, RefCurv was also used for constructing age- and sex-specific percentile charts and subsequent z-transformation of cortisol levels.

For constructing percentile charts, RefCurv relies on the LMS method, which assumes that a distribution of data can be normalized by Box-Cox transformation. The three parameters L (λ, skewness of distribution), M (μ, median) and S (σ, coefficient of variation) for Box-Cox transformation were selected by choosing the subset of hyperparameters providing the lowest Bayesian Information Criterion (BIC) after grid search considering 5 degrees of freedom (df) for each hyperparameter (30). Model verification was performed by cross-validation as implemented in RefCurv. The best model fit (weighted against overfitting) was provided by 0 dfs for the (penalized) splines of λ, μ, and σ in boys as well as in girls.

Before constructing reference curves, data of the norming sample were semi-winsorized, a process by which a pre-defined value replaces outliers (31). In the present study, outliers were defined as cortisol levels exceeding ± 3 SDs considering age and sex and were replaced with a value corresponding to ± 3 SDs. Thus, 1.1% of raw data were winsorized before analysis with RefCurv, which is well below a recommended threshold of 5% (31).

Elevated cortisol and TSH levels were defined by a z-standardized value above 1.96 SD (> 97.5th percentile) considering age and gender.

### Statistical Analysis

Data handling and statistical analyses were performed with SPSS 26.0 (Armonk, NY: IBM Corp.) as well as R (version 4.0.3, R Core team, 2020) and the ‘quantreq’ [Version 5.75 (32)] and ‘partialloverlapping’ [Version 2.0 (33)] package.

*H_1A_* and *H_1B_* were assessed by two-tailed testing and results were considered significant at *p* <.05. However, considering the conflicting nature of hypotheses *H_1C_*– *H_2B_, *results related to testing these hypotheses were corrected for multiple comparisons by a Bonferroni correction (*p_crit_* <.008). Regarding those methods that do not provide a *p*-value but only bootstrapped confidence intervals (CI), the α-level for determining the CI was adjusted accordingly.

Effect size calculations [*d* (small 0.20 ≤ d ≤ 0.49, medium 0.50 ≤ *d* ≤ 0.79, large ≥ 0.8)] relied on SPSS or an online calculator (34). Power analyses were performed with GPower 3.1 (35) (assuming α = .05, two-tailed testing, sufficient power = 1-β ≥ 0.8), but there is currently no option to perform power analysis for quantile regression as implemented in the present study.

Normality of the distribution of the continuous variables of interest was assessed by the Shapiro-Wilk test and visual inspection of Q-Q plots. Outliers (exceeding 3 times the interquartile range) were identified by box plots. Non-normally distributed variables were transformed according to Templeton (36), preserving the mean and standard deviation of the transformed variable.

#### Sample Characteristics

Considering dependencies between samples, an exploratory analysis of study characteristics between the total sample and two subsamples of patients was performed by either t-tests (continuous variables) or tests of proportions (categorical variables) as implemented in the ‘partiallyoverlapping’ package. The first subsample included patients *with* and the second subsample only those patients *without* medication affecting endocrine functioning (psychotropic drugs and COC).

#### Mean Comparisons

One-sample t-tests against zero were employed to compare z-standardized cortisol (z-cortisol), TSH (z-TSH) and fT4 (z-fT4) to the respective norming sample (mean (*M*) = 0, standard deviation (*SD*) = 1) used for assay calibration.

Information regarding the intake of medication was not available for the reference sample. However, to consider the potential effects of COC usage and psychotropic medication on endocrine functioning, z-cortisol, z-TSH, and z-fT4 were also compared between the total sample and a subsample of patients free of medication. Also, the potential effects of inpatient and outpatient status on cortisol levels were compared between both groups by a two-sample t-test. Note, these analyses were deemed exploratory as they were only conducted to verify the main findings of the present study.

#### Quantile Regression

The commonly applied ordinary least square (OLS) regression approach is a regression about the mean that assumes a uniform relationship between the dependent variable and a predictor variable across the full range of the distribution of the dependent variable conditional on the levels of the predictor variable. Quantile regression allows for relaxing this assumption and for determining slope coefficients for different quantiles of the dependent variable. For example, in the case of the 20^th^ percentile (τ = 0.2), this would be the relationship between the predictor and the lowest 20 percent of values of the dependent variable. In addition to this advantage of quantile regression to explore the relationship between the dependent variable and a predictor beyond the mean, quantile regression has several advantages over OLS regression. Amongst others, quantile regression is semiparametric as it does not make any assumptions regarding the distribution of the error term of the model. Moreover, quantile regression is reasonably robust to outliers regarding the dependent variable (37).

Considering the stated hypotheses, the linear relationship between z-TSH, z-fT4, and z-cortisol was studied at two quantiles of the distribution of z-cortisol levels. First, at the 50^th^ percentile (τ_z-cortisol_ = 0.5) and second, at a percentile indicating pathologically elevated cortisol levels, i.e., a z-score of 1.96 (τ_z-cortisol_ = z_1.96_).

Due to a large number of potential covariates [age, sex, z-BMI, smoking, use of COC, psychotropic medication, 25(OH)-vitamin D levels, presence of TPO antibodies (TPO-AB), BDI-II score (depression severity)], a subset was selected by considering only those variables with a significant correlation with either outcome measure (cortisol, TSH, and fT4) (38). Correlation analyses were performed considering the scale of measure (interval scaled variables: Pearson correlation *r*; interval scaled and dichotomous variable: point-biserial correlation *r_pb_*) as indicated. No correction for multiple comparisons was applied concerning this step of analysis as the H_0_ (*no* significant correlation) was the favored outcome. To avoid problems with (matrix) singularity that is not readily dealt with by the ‘quantreg’ package, categorial covariates significantly correlated with either z-TSH, z-fT4, or z-cortisol (sex, smoking status, psychotropic medication, COC, s. **Table 2**) were partialled out of all other variables of interest, including continuous covariates.

**Table 2:** Bivariate correlations.

	z-TSH	z-fT4	TPO-AB	z-cortisol	age	z-BMI	BDI-II score	25(OH)-vitamin D	sex	smoking	COC	psychotropic medication
z-TSH	1											
z-fT4	.02	1										
TPO-AB	.24**	-.07	1									
z-cortisol	.10*	.02	-.04	1								
age	-.03	.14*	-.03	-.15**	1							
z-BMI	.16**	.003	.09	-.04	-.09	1						
BDI-II score	-.07	-.05	-.01	.04	.02	.01	1					
25(OH)-vitamin D	-.03	.03	.03	-.08	-.03	-.09	.12*	1				
sex	-.05	-.19	-.01	.24**	-.08	-.07	.25**	.10*	1			
smoking	-.13**	.03	-.20**	-.05	.14**	-.03	.13**	.03	-.07	1		
COC	.03	.03	-.02	.20**	.19**	-.07	.01*	.001	.17**	-.06	1	
psychotropic medication	-.01	-.16**	-.09	-.09	.06	.01	.08	.06	.04	06	-.05	1

Pearson correlation or point-biserial correlation, depending on the scale of measure, for the total sample; z, z-standardized. *p < .05, **p < .01.

Quantile regression models were fitted relying on a modified simplex algorithm adapted by Koenker et al. (39). Regarding central quantiles in the range between the 30^th^ and 70^th^ percentile, a Huber sandwich estimator was employed to determine standard errors. However, considering the sample size as well as the number of IVs, a bootstrapping subsampling method suggested by Chernozhukov et al. (40) was used to compute standard errors in the range of more extreme quantiles following recent recommendations by the same authors.

Quantile regression coefficients at the above-outlined quantiles were compared by a Wald test to assess hypotheses *H_2A_* **** and **** *H*_*2B*_ and homoscedasticity. Outlier detection relied on boxplots as detailed above, and linearity between continuous variables was checked by visual inspection of bivariate scatter plots.

*R1* was determined to provide a local measure of goodness of fit at the quantiles outlined above. While *R^2^* in OLS regression quantifies the amount of variance accounted for in the dependent variable, *R1* quantifies the weighted sum of absolute residuals accounted for in the dependent variable in quantile regression and is, likewise *R^2^*, bound between 0 and 1 (41). Against these considerations, the size of *R1* is interpreted tentatively, with all due caution, like *R^2^*.

## Results

### Sample Characteristics

The mean age of the 273 patients was 15.9 (*SD* 1.5) years and the mean BDI-II score 30.3 (*SD* 11.5), indicating severe depressive symptoms. Altogether, 91.6% of adolescents experienced a first depressive episode and 22.7% were taking psychotropic medication including antidepressants on admission. Elevated (z > 1.96) z-TSH scores were observed in 8.1% and elevated z-cortisol scores in 17.6% of patients (for detailed descriptive statistics, see **Table 3**).

**Table 3:** Patient characteristics.

	Adolescents with MDD (N = 273)	Medication-free MDD subsample (N = 191)	Adolescents with MDD and medication (N = 58)
age	15.85 (1.52)	15.64 (1.53)^$^	16.35 (1.39)°
	[11.80 - 18.83]	[11.80 - 18.83]	[12.71 - 18.89]
gender (female %)	76.2	72.8^$^	84.1°
z-BMI	0.18 (1.46)	0.19 (1.46)	0.17 (1.44)
	[-4.93 - 3.09]	[-4.93 - 2.76]	[-4.45 - 3.09]
BDI-II	30.32 (11.47)	29.31 (11.02)^$^	32.67 (12.20)°
	[2 - 60]	[2 - 59]	[7 - 60]
first depressive episode (%)	91.6	92.1	87.8
psychotropic medication (%)	22.7	–	75.6
smoking (%)	19.8	19.4	20.7
combined oral contraceptives (%)^#^	11.5	–	29.3
TSH (mU/l)	2.38 (1.48)	2.37 (1.61)	2.39 (1.13)
	[0.56 - 18.65]	[0.56 - 18.65]	[0.74 - 6.14]
z-TSH	0.69 (0.89)	0.67 (0.92)	0.75 (0.83)
	[-8.22 - 4.10]	[-1.99 - 4.10]	[-1.37 - 2.54]
z-TSH > 97.5 percentile (%)^§^	8.1	7.3	9.8
fT4 (pmol/l)	14.25 (2.05)	14.45 (2.09)	13.77 (1.87)
	[9.5 - 20.1]	[9.9 - 20.1]	[9.5 - 19.8]
z-fT4	-0.12 (1.01)	-0.02 (1.03)^$^	-0.36 (0.93)°
	[-2.57 - 2.64]	[-2.35 - 2.64]	[-2.57 - 2.53]
TPO-AB positivity (%)	4.8	4.7	4.9
cortisol (nmol/l)	485.27 (208.40)	462.12 (174.76)	539.18 (264.57)
	[26.11 - 1557.37]	[26.11 - 1117.22]	[120.81 - 1557.37]
z-cortisol	1.22 (0.84)	1.17 (0.87)	1.33 (0.96)
	[-2.99 - 4.25]	[-2.99 - 3.59]	[-1.04 - 4.25]
z-cortisol > 97.5 percentile (%)^§^	17.6	15.7	22.0
25(OH)-vitamin D (nmol/l)	34.45 (15.62)	34.04 (15.45)	35.41 (16.05)
	[11.23 - 86.36]	[11.23 - 86.36]	[11.73 - 76.38]
25(OH)-vitamin D < 12 nmol/l (%)	46.2	45.5	47.6

Mean, standard deviation (in round brackets), and range (in square brackets) for interval scaled variables and percentages otherwise, separately for the total sample and the subsamples of patients with and without the intake of COC and/or psychotropic medication; z, z-standardized. ^$^significant difference between the medication-free subsample and the total sample (p_uncorected_ < .05), °significant difference between the subsample on medication and the total sample (p_uncorected_ < .05), ^#^percentage only regarding women, ^§^percentiles refer to the reference sample used for assay calibration.

Prior to a comparison of characteristics between the total sample and the subsamples of patients with and without medication affecting endocrine functioning, age and 25(OH)-vitamin D were rank-transformed to account for non-normality.

Patients free of medication were younger (*t*(238.07) = 3.60, *p* = <.001, *d* = 0.47) and less depressed (*t*(238.07) = 2.26, *p* = .03, *d* = 0.29), patients on medication were older (*t*(227.34) = -3.62, *p* = <.001, *d* = 0.48) and more depressed (*t*(227.34) = -2,19, *p* = = .03, *d* = 0.29) than patients of the total sample, but absolute differences were small. Except for a slightly but also significantly smaller proportion of female patients in the medication-free subsample (*z* = 1.98, *p* = .047), there were no other differences in study characteristics compared to the total sample. In patients on medication, there was a significantly higher percentage of female patients than in the total sample (*z* = -2.09, *p* = .04).

### T-Tests (*H_1A_* and *H_1B_*)

**Figure 1:**
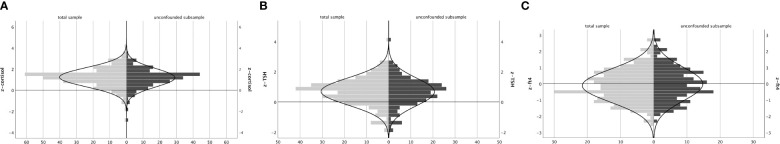
Frequencies of z-standardized **(A)** cortisol, **(B)** TSH, and **(C)** fT4 levels with an overlaid normal distribution curve, separately plotted for the total sample and the subsample of patients without the intake of medication affecting endocrine functioning. The horizontal line indicates z = 0 and, therefore, the mean of the reference sample used for calibration of the respective hormone assay.

In MDD, rank-transformed z-cortisol (z-cortisol_trans_; *t*(272) = 22.38, *p* = <.001, *d* = 1.35, **Figure 1**) and z-TSH (*t*(272) = 12.84, *p* = <.001, *d* = 0.79) were found to be significantly elevated in comparison to the respective norming sample (**** *H_1A_*disproved). In contrast, z-fT4 levels were significantly lower in MDD than in the norming sample (*t*(272) = - 1.99, *p* = .047, d = 0.12; *H_1B_* **** disproved).

There was no difference in z-cortisol_trans_ (subsample_medication-free_: *t*(238.07) = 1.12, *p* = .26; subsample_medicated_: *t*(227.34) = -1.07, *p* = .29) and z-TSH (subsample_medication-free_: *t*(238.07) = 0.70, *p* = .48; subsample_medicated_: *t*(227.34) = -0.72, *p* = .47) between the two subsamples and the total sample. However, in patients with and without medication, z-fT4 was significantly lower (*t*(227.34) = - 2.56, *p* = .01, *d* = 0.34) and higher (*t*(238.07) = -2.50, *p* = .01, *d* = 0.32), respectively, than in the total sample. Moreover, in those depressed adolescents on medication, z-fT4 levels were lower than in the norming sample (subsample_medicated_: *t*(81) = -3.46, *p* <.001). Thus, even though these findings suggest that z-fT4 levels in MDD were affected by the intake of medication, there is yet no evidence of a relationship between cortisol and z-fT4 in line with *H_1B_* proposing increased z-fT4 levels in MDD.

No significant difference in z-cortisol_trans_ was found between inpatients (*M* = 1.05, *SD* = 0.94) and outpatients (*M* = 1.40, *SD* = 0.82; *t*(92) = - 1.44, *p* = .154). However, power was only sufficient to identify large mean differences between both groups of patients (power (1-β) = 0.84 for *d* = 0.8 but 0.45 for *d* = 0.5).

### Quantile Regression (*H_1C-D_*, *H_2A-B_*)

**Figure 2:**
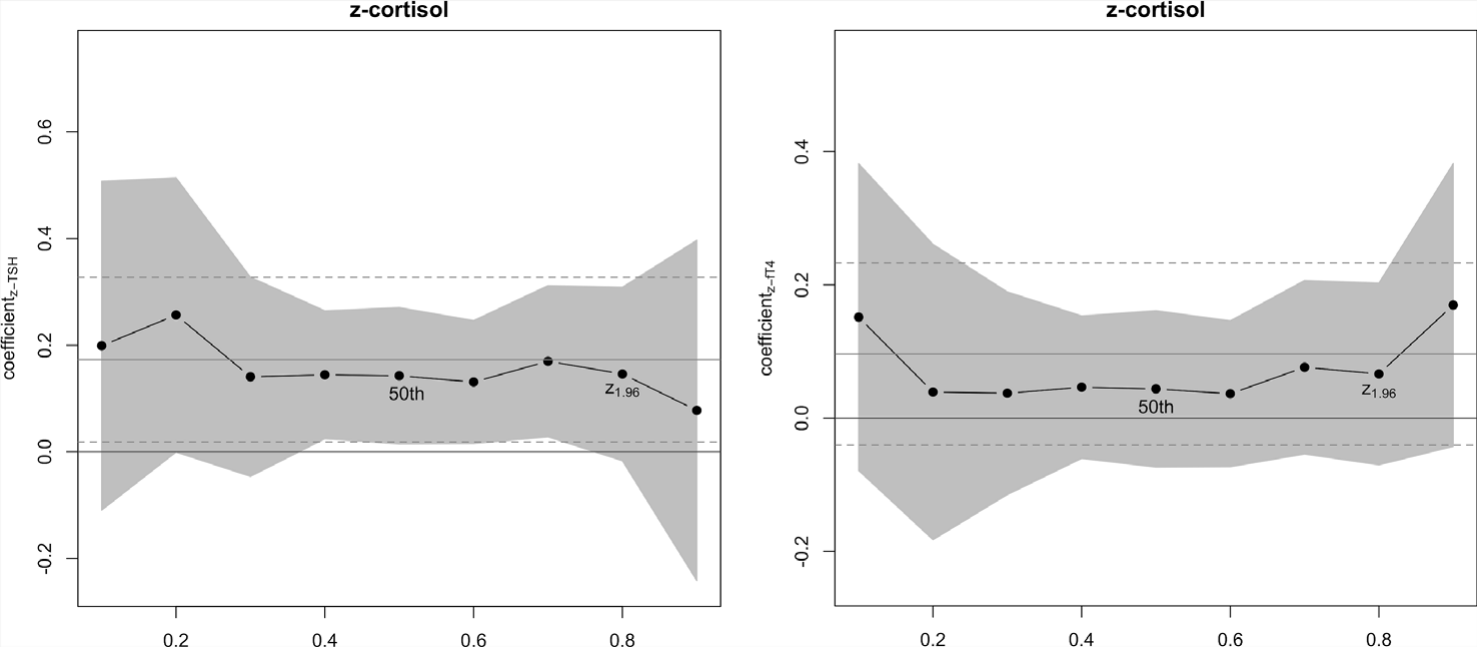
Quantile regression coefficients (y-axis) for different quantiles (τ, x-axis) regarding the relationship between z-standardized TSH, fT4, and cortisol. A 99.2% confidence interval (CI), resulting from adjustments for multiple comparisons, for the quantile regression coefficients at the respective τ-levels is displayed in dark grey (Note, only the CI based on the standard error derived from the Huber sandwich estimator is displayed). Horizontal solid lines in light gray correspond to the multiple regression coefficient on the y-axis of the respective figure, and horizontal dashed lines in light grey represent the 99.2%-CI regarding these coefficients. The 50^th^ percentile and the percentile at which z equals 1.96 are highlighted.

While there was no significant difference between the quantile regression coefficients at τ_z-cortisol_ = 0.5 and τ_z-cortisol_ = z_1.96_ regarding z-TSH (*F*(1, 545) = 0.003, *p* = .95; **Table 4** and **Figure 2**), there was a significant and positive relationship between z-TSH and z-cortisol_trans_ at τ_z-cortisol_ = 0.5 (*b* = 0.14, *t*(266) = 2.96, *p* = .003, *R1* = 0.016, *d* = 0.25) but not at τ_z-cortisol_ = z_1.96_ (*b* = 0.15, 95%-CI [-0.49; 0.13], *R1* = 0.018; *H_1C_* **** **disproved, *H_2A_* ****disproved). For neither quantile, z-cortisol_trans_ was related to z-fT4 (τ_z-cortisol_ = 0.5: *b* = 0.04, *t*(266) = 0.99, *p* = .32, *R1* = 0.003; τ_z-cortisol_ = z_1.96_: *b* = 0.07, 95%-CI [-0.48; 0.21], *R1* = 0.004; *H_1D_* **** **disproved), and there was no significant difference between the quantile regression coefficients at τ = 0.5 and τ = z_1.96_ regarding z-fT4 (IV; *F*(1, 545) = 0.22, *p* = .64; *H_2B_* ****disproved).

**Table 4:** Results - quantile regression.

quantile		τ_z-cortisol_ = 0.5				τ_z-cortisol_ = z_1.96_	
	b	SE	t	p	b	95%-CI_lower_	95%-CI_upper_
age	-0.13	0.03	-4.25	<.001	-0.10	-0.68	0.16
z-BMI	-0.05	0.03	-1.80	.07	0.02	-0.62	0.07
25(OH)-vitamin D	0.005	0.004	1.48	.14	0.01	-0.39	0.10
TPO-AK	0.001	0.001	0.98	.33	0.0004	-0.38	0.07
z-fT4	0.04	0.04	0.99	.32	0.07	-0.48	0.21
z-TSH	0.14	0.05	2.96	.003	0.15	-0.49	0.13

τ, quantile; b, regression coefficient; SE, standard error; t, t-value; p, p-value; CI, confidence interval; z, z-standardized.

## Discussion

In adults, it is acknowledged that HPA axis dysfunction is a major endocrine cause of HPT axis dysfunction, but this has not yet been investigated in adolescent MDD. In contrast to previous findings in adults, in adolescent MDD in the present study, TSH levels were found to be elevated and fT4 levels unchanged. Moreover, at the median of cortisol levels, a positive linear relationship between cortisol and TSH but not fT4 was observed, which likewise conflicts with predictions from the hypothesized pathophysiological framework relating HPA and HPT axis functioning in MDD in adults. These findings imply that both axes are distinctively regulated in adolescent and adult MDD. Moreover, when also considering the small effect size of the relationship between TSH and cortisol that is only present at the median of cortisol levels, the results of the current study suggest that in adolescent MDD, the HPA and HPT axis are likely only loosely related and may very well function independently in the pathological range of hormone levels.

### HPA and HPT Axis Functioning in Adolescent Depression

In the present study, cortisol levels were found to be elevated in adolescent MDD. This observation has also previously been made, but in contrast to a large effect size in the present study, a meta-analysis indicated only a borderline effect size regarding the impact of MDD on cortisol levels (*d* = 0.2) (42). As discussed by the authors of the meta-analysis, there was a notable heterogeneity of findings with limited generalizability across studies, likely due to inconsistencies in the measurement of basal cortisol levels as well as small and heterogeneous samples by individual studies. These limitations may contribute to an explanation of a large effect size of MDD on cortisol levels in the present study and an aggregated, small effect size by previously conducted studies.

While the authors of the aforementioned meta-analysis did not find a difference between children adolescents and adolescents adults regarding HPA axis functioning (42), a subsequent review suggested a confounding effect of age on cortisol levels (43). This putative effect of age on the functioning of the HPA axis is supported by another review examining the endocrine system in child and adolescent depression in comparison to adults (44). As highlighted by the authors of the latter review, maturational adjustments of the neurotransmitter system and increasing ramifications of illness duration and comorbidities that occur with progressing age may explain differences between adolescents and adults regarding endocrine functioning in MDD. Thus, age may not only affect the HPA axis but also its interplay with the HPT axis. This conclusion is rather plausible when considering the hypothesized pathophysiological framework as subsequently shortly summarized with its implications for the results of the present study.

In adult depression, endocrine dysfunction of the HPT axis has been related to increased cortisol levels (1, 3), which are hypothesized to alter hippocampal functioning and, thereby, decrease TSH release from the pituitary gland (5, 6). The hypothesis of cortisol-induced neurotoxicity due to long-term elevated cortisol levels in MDD (45) has previously been investigated and is supported by meta-analyses of cross-sectional volumetric MRI studies, which either evidenced reduced hippocampal size in depressed adults with a depression duration of at least 2 years (46) or more severe hippocampal volume reductions in those with more long-standing depression (47). Considering these findings, hippocampal size and potentially also hippocampal functioning are likely unaffected in patients with a first episode of MDD, which applied to 91.6 percent of adolescents in the present study. Thus, effects of age and illness duration likely explain differences between adolescents and adults with MDD concerning the interplay of the HPA and HPT axis.

However, concerning the hypothesized effect of HPA axis functioning on the HPT axis, it is important to note that there is a lack of studies providing causal support of the above-outlined pathophysiological framework of cortisol-induced hippocampal neurotoxicity. By now, there is no evidence from longitudinal MRI studies indicating that cortisol levels in MDD might be associated with (smaller) hippocampal volumes (45, 48). Moreover, HPA axis dysfunction in MDD may affect TRH secretion to a lesser extent than previously thought. Two previous studies reported increased TRH levels in the CSF of depressed patients (11, 12), but these studies included only 12 to 15 patients. Moreover, this finding could not be replicated in four independently conducted studies (15–18). Instead, there is evidence of decreased TRH gene expression in hypothalamic post-mortem specimens of patients with MDD, even though conclusions are limited by a sample size of 7 patients (49). Thus, also other mechanisms than HPA axis-related dysfunction of the HPT axis may have to be considered to explain altered thyroid function test results in adults with MDD. In this regard, it is important to note that previous evidence that dysfunction of the HPT axis may result in altered functioning of the HPA axis and hypercortisolemia (50, 51) has not been replicated by several studies in animals (52) and men (53–55). Rather, as discussed by Kirkegaard et al. (56) in detail, abnormal serotonin and noradrenaline signaling may be related to dysfunction of the HPT but also the HPA axis. Alternatively, Kutcher et al. (21) suggested that abnormalities of the HPT and HPA axis may most parsimoniously be explained by altered somatostatin levels, which is consistent with growing evidence of somatostatin deficiency in MDD (57). Considering age-dependent changes of these neurotransmitter and hormone systems similar to those outlined above regarding HPA axis functioning (44, 58), maturational aspects may also explain different thyroid function test results between depressed adolescents and adults observed not only in the present study but also by previous research (13, 21).

### Limitations

As in the norming sample (24), serum cortisol was measured in the early morning by a single point measurement. It has been debated whether this approach allows for a reliable assessment of HPA axis functioning, but a study addressing this issue found high intra-individual stability of single point serum cortisol levels over a period of more than two years, likely because there is a genetically determined set-point for HPA axis activity (59).

In the present study, pathology of the HPA axis was defined by hypercortisolemia, but no functional testing (e.g., dexamethasone suppression test) was performed to further investigate dysfunction of the HPA axis. Likewise, regarding the functioning of the HPT axis, TSH levels but no functional tests have been used (e.g., TRH stimulation tests). However, TSH levels have been found to be intra-individually stable (60), especially in patients with (subclinical) dysfunction of the HPT axis (61). Therefore, further testing of the HPT axis is likely not necessary to determine dysfunction, especially when also considering significant side effects related to its assessment by the TRH test (62).

Additionally, the anticipation of venipuncture and also hospitalization may activate the HPA axis in a substantial but unpredictable number of subjects (63) which may result in elevated cortisol levels unrelated to the underlying condition. However, in the present study. there was no evidence of an effect of hospitalization on cortisol levels when comparing inpatients and outpatients. Moreover, according to the central limit theorem, the risk of oversampling individuals with heightened cortisol reactivity, for example, in anticipation of venipuncture, reduces with more than 30 observations, and this is supported by the distribution of cortisol levels in the present study (**Figure 1**).

When comparing cortisol levels between the norming sample and the sample of adolescents with MDD, we could not account for the usage of COC or intake of psychotropic medication as this information was not available for the norming sample. However, in the sample of adolescents with MDD, COC usage explained only 4% of the variance in cortisol levels, and there was no difference between cortisol levels in the total sample and the subsample of patients without medication affecting HPA axis functioning.

Considering an analysis of cross-sectional data in the present study, there is a need for a longitudinal study, at best relying on cumulative measures of HPT axis functioning, to causally relate the HPA and HPT axis in child and adolescent MDD, either in those patients at risk for depression or in the follow-up of a (first) depressive episode.

### Conclusions

This is the first study to address the relationship between the HPT and HPA axis in a large, cross-sectional sample of adolescents with MDD with sufficient power to identify even small effects concerning their interplay. Findings from our study point to dysfunction of both, the HPA and HPT axis in adolescent MDD as indicated by elevated cortisol and TSH levels in comparison to healthy controls. Moreover, in contrast to adults, there was no evidence of a substantial relationship between the two endocrine systems, neither in patients with normal nor in patients with elevated cortisol levels.

## Data Availability Statement

The raw data supporting the conclusions of this article will be made available by the authors, without undue reservation.

## Ethics Statement

The studies involving human participants were reviewed and approved by Ethics Committee of the University Hospital Essen, University of Duisburg Essen (No. 15-6363-55 BO). Written informed consent to participate in this study was provided by the participants’ legal guardian/next of kin.

## Author Contributions

RH conceptualized the present study, analyzed and interpreted the data, and wrote the manuscript. CG, MF, LL, JA, AH, JB, JH and RH designed and undertook the clinical studies and collected the data. PMH and AK obtained the data of the normative sample. All authors contributed to the article and approved the submitted version.

## Funding

RH was supported by the UMEA Clinical Scientist Program by the Faculty of Medicine of the University of Duisburg-Essen and the German Research Foundation (DFG). The funders had no role in the study design, data collection and analysis, decision to publish, or preparation of the manuscript.

## Conflict of Interest

The authors declare that the research was conducted in the absence of any commercial or financial relationships that could be construed as a potential conflict of interest.
